# Substance Use and Mild Traumatic Brain Injury Risk Reduction and Prevention: A Novel Model for Treatment

**DOI:** 10.1155/2012/174579

**Published:** 2012-05-17

**Authors:** Jennifer H. Olson-Madden, Lisa A. Brenner, John D. Corrigan, Chad D. Emrick, Peter C. Britton

**Affiliations:** ^1^Mental Illness Research, Education, and Clinical Center (MIRECC), Eastern Colorado Health Care System (ECHCS) Veterans Affairs Medical Center, Denver, CO 80220, USA; ^2^Department of Psychiatry, School of Medicine, University of Colorado Denver, Aurora, CO 80111, USA; ^3^Departments of Neurology and Physical Medicine and Rehabilitation, School of Medicine, University of Colorado Denver, Aurora, CO 80111, USA; ^4^Department of Physical Medicine and Rehabilitation, Wexner Medical Center at The Ohio State University, Columbus, OH 43210, USA; ^5^Outpatient Substance Abuse Treatment Program, Eastern Colorado Health Care System (ECHCS) Veterans Affairs Medical Center, Denver, CO 80220, USA; ^6^Center of Excellence, Canandaigua Veterans Affairs Medical Center, Canandaigua, NY 14424, USA; ^7^Department of Psychiatry, University of Rochester Medical Center, Rochester, NY 14642, USA

## Abstract

Traumatic brain injury (TBI) and substance use disorders (SUDs) frequently co-occur. Individuals with histories of alcohol or other drug use are at greater risk for sustaining TBI, and individuals with TBI frequently misuse substances before and after injury. Further, a growing body of literature supports the relationship between comorbid histories of mild TBI (mTBI) and SUDs and negative outcomes. Alcohol and other drug use are strongly associated with risk taking. Disinhibition, impaired executive function, and/or impulsivity as a result of mTBI also contribute to an individual's proclivity towards risk-taking. Risk-taking behavior may therefore, be a direct result of SUD and/or history of mTBI, and risky behaviors may predispose individuals for subsequent injury or continued use of substances. Based on these findings, evaluation of risk-taking behavior associated with the co-occurrence of SUD and mTBI should be a standard clinical practice. Interventions aimed at reducing risky behavior among members of this population may assist in decreasing negative outcomes. A novel intervention (Substance Use and Traumatic Brain Injury Risk Reduction and Prevention (STRRP)) for reducing and preventing risky behaviors among individuals with co-occurring mTBI and SUD is presented. Areas for further research are discussed.

## 1. Introduction

Traumatic brain injury (TBI) and substance use disorders (SUDs) frequently co-occur. Individuals with histories of alcohol or other drug use are at greater risk for sustaining TBI, and individuals with TBI frequently misuse substances pre- and post-injury [1–6]. Research suggests that members of general population who consume alcohol are at four times the risk of sustaining a TBI than those who do not [2]. Up to 75% of TBIs are incurred when individuals are intoxicated [2, 7]. These figures are not surprising given that alcohol use is implicated as a risk factor for injury resulting from motor vehicle accidents, falls, and/or violence. Further support for the link between intoxication and serious injury exists due to factors such as poor motor control, impaired decision making, vulnerability to victimization, or propensity toward belligerent/aggressive behaviors secondary to substance use. Moreover, prior history of a SUD, regardless of the presence of intoxication at time of injury, is a risk factor for morbidity and excessive use following injury [8].

Postinjury substance using behaviors are also problematic [9–11]. While a decrease in alcohol and other drug use and higher rates of abstinence have been observed immediately after TBI [12, 13], return to preinjury levels of consumption [12, 13] or increased use at one year after injury have been reported [14, 15]. Long-term substance abuse may increase as the time postinjury increases [9, 14–16], particularly among individuals whose use is not restricted by external factors (e.g., living in an institution where access to substances is limited, under consistent supervision by a caregiver who does not use substances). In the Veteran population, Brenner and colleagues [17] found that among individuals with TBI, the likelihood of problematic postinjury drug and alcohol use, given a preinjury history, was significantly higher than the probability given no history. Similar results were observed for members of the general TBI population with a history of alcohol abuse [13].

A growing literature supports the relationship between comorbid histories of TBI and SUDs and negative outcomes [5, 8, 15]. Those with co-occurring histories are at greater risk for subsequent injury and/or psychosocial and psychiatric problems than those without such misuse [1, 3, 18–21]. In comparison to those who sustained a TBI but did not have substance use problems, individuals with histories of co-occurring misuse/abuse and TBI report (1) lower subjective well being and life satisfaction; (2) unmet psychological needs; and (3) increased perceived barriers to mental healthcare [22–24]. Alcohol use prior to sustaining a TBI has been found to increase the risk of an individual developing mood disorders after injury [22], and individuals with substance abuse (SA) after TBI exhibit more severe psychiatric symptoms than those who have SA problems without a history of TBI. Additionally, in comparison to the general population, the risk of death by suicide has been reported as being four times higher for those with TBI and co-morbid SUD [25]. Olson-Madden and colleagues [26] found that, among SA treatment-seeking Veterans with a history of TBI, individuals at risk for TBI are also at risk for mental illness and vice versa, providing further support for the hypothesis that the cumulative impact of co-occurring conditions would be expected to culminate in poorer outcomes [26]. 

Risk taking refers to the tendency to engage in behaviors that have the potential to be harmful or dangerous, but which may be perceived by the person engaging in the behavior as an opportunity to obtain a positive outcome (e.g., short-term pleasure). Such risk taking is present in a variety of behaviors, including substance use, gambling, unprotected sex, or dangerous pasttimes such as skydiving. Risky behavior can be conceptualized as an expression of a personality trait that Zuckerman [27] identifies as “sensation seeking.” This trait embodies four components: thrill/adventure seeking, experience seeking, disinhibition, and susceptibility for boredom. Zuckerman [28] conceptualizes this trait as having a strong biological influence, claiming that a tendency toward sensation seeking and risk taking may be genetic.

Another factor that can contribute to risky behavior is an individual's appraisal of risk. Work by Gilman and colleagues [29] shows that after consuming alcohol, social drinkers have decreased sensitivity in brain regions involved in detecting threats and increased activity in brain regions involved in reward [29]. This suggests that after alcohol exposure, threat-detecting brain circuits are less able to differentiate between threatening and nonthreatening social stimulus. One outcome could be failure to avoid risky situations (e.g., an argument and a fight). Dulled reactions also could be translated into potentially dangerous situations such as drunk driving.

Studies show that there is a strong association between risk taking and alcohol and other drug use [27]. The search for novel experiences sets off the same brain reward system as substance use, providing a biological explanation for alcohol and drug abuse among people who constantly seek out new and exciting experiences. The hypothesis is that the inhibitory response of these personality types is also diminished, adding to the individual's tendency to take risks. Disinhibition, impaired executive functioning, and/or impulsivity (e.g., lack of premeditation and sense of urgency) [29–31] as a result of TBI could also contribute to an individual's proclivity towards risk taking. Risk-taking behavior may be a direct result of SA and/or TBI, and risky behaviors may predispose individuals for subsequent injury or continued use of substances. As such, evaluation of risk-taking behavior associated with the co-occurrence of SA and TBI seems to be a salient area for further study.

## 2. Implications for Treatment

 Critical gaps exist with regard to how best improve TBI-related outcomes for individuals who continue to engage in risky behaviors like misusing substances. According to the Centers for Disease Control Injury Research Agenda [32], priorities regarding TBI intervention should focus on understanding and preventing the development of secondary conditions following TBI, and identifying strategies to ensure care for those with TBI. Some findings suggest that meeting the needs of individuals with SUDs and TBI may require clinicians to modify current practices to address cognitive dysfunction, poor emotional regulation, and/or limited social skills. For those with mild TBI (mTBI), few evidence-based treatments are available. Generally, comprehensive neuropsychological rehabilitation therapies, which involve a combination of therapies targeting cognitive, emotional, interpersonal, and motivational deficits associated with TBI, are focused on, and more effective for, those with moderate to-severe TBI. While there is some evidence that rehabilitation increases community integration and engagement in work in persons with moderate/severe TBI, the same has not been demonstrated in individuals with mTBI [33]. Given the unique needs of individual with mTBI and co-occurring SUDs, treatments focusing on providing education regarding symptoms of both conditions, coping strategies to minimize their impact, and reducing risky behaviors may be more successful than existing treatments [34].

There is also some evidence that motivational interviewing (MI) techniques and motivational counseling [35] may be potentially efficacious for preventing use or return to substance abuse postinjury. Similarly, structured motivational counseling [36], a community model using consumer and professional education, case management and consultation to address SUDs in adults with mTBI, also may improve outcomes. Quasiexperimental studies have provided modest support for the efficacy of MI and case management among those with history of TBI. Furthermore, there is some evidence to suggest that increasing negative outcome expectancies could lead to reductions in drinking [37]. As such intervention strategies which increase negative substance expectancies may contribute to risk reduction. Specifically, motivational enhancement therapy (MET) [36] may assist clients in exploring the pros and cons of substance abuse, or the pros and cons of thrill seeking or other risky behavior.

 Employing such strategies may have particular relevance for individuals at elevated-risk for mTBI and SUDs, such as military personnel returning from conflicts. In particular, among Operation Enduring Freedom/Operation Iraqi Freedom Soldiers (OEF/OIF), TBI has been described as the signature wound, with rates of mTBI being reported as high as 23% [38]. The RAND Corporation [39] reported a “probable” TBI prevalence of 19.5%, which is equated with approximately 320,000 Veterans who served in Iraq or Afghanistan having sustained probable TBIs. Furthermore, a recent study by Olson-Madden and colleagues [26] indicated that 55% of a sample of Veterans seeking SA treatment in a metropolitan VA hospital had a positive history of TBI. Significant rates of SUDs among returning Military Personnel have also been identified (e.g., 11% acknowledge having an alcohol problem) [40]. 

## 3. A Potential Intervention to Address Risky Behavior

Individuals with mTBI and SUD may warrant a treatment targeting their potentially unique needs. The Substance Use and Traumatic Brain Injury Risk Reduction and Prevention (STRRP), seeks to address the tension between preventing a behavior and reducing the harmfulness of that behavior (e.g., harm reduction and risk reduction). It is an integrative model that incorporates aspects of motivational enhancement treatments as well as more educational approaches to treating individuals with mTBI and SUD. Specifically, individuals are provided with information and then asked to explore the relevance of this information using ME strategies. One example of this is providing information regarding common difficulties after mTBI which is offered at the beginning of one session, and group members are invited to consider how such problems might manifest in their own lives. They are then invited to consider the implications of reducing or avoiding behaviors that may contribute to, or be exacerbated by, those problems. The underlying therapeutic principles in STRRP include empathic listening, developing and emphasizing the discrepancy between the individual's present behavior and his/her goals and values, and supporting self-efficacy and confidence to change risky behaviors. A consistent finding in the literature involves the impact of therapist characteristics [41], with therapist empathy as a significant predictor of favorable treatment outcomes [42, 43]. Given the lack of knowledge concerning common manifestations and consequences of mTBI, educational components include a review of the common manifestations of mTBI, as well as a focus on symptom management, including physical, cognitive, and behavioral/emotional sequelae.

Specifically, it combines evidence-based guidelines with an orientation toward recovery that values clients' personal experiences and choices [44]. While this intervention and accompanying manual was designed to be used within the high-risk population of those with a history of mTBI seeking SA treatment, reducing alcohol and other drug use behaviors are not the only targets for intervention. The intervention emphasizes the importance of decreasing all behaviors that are harmful and increase an individual's risk for poor outcomes including risk for reinjury and psychiatric/psychosocial symptoms. It was developed based on the premise that changing risky behaviors in general is likely to result in positive SUDs treatment gains and reduce the risk for future TBI and other negative outcomes.

STRRP is based largely on the premise that people need to be ready for change [45]. This model suggests that individuals move through five stages of change, from not thinking about change to maintaining long-term change. This approach requires that the clinician continually assesses the client's readiness to change and promotes motivation through a series of techniques based on that individual's level of readiness. In particular, MI [19] and group-based ME [36] strategies are integral to each STRRP session for the purpose of facilitating clients' movement toward decreasing risky behaviors. MI is a well-documented approach informed by the Stage Theory of Behavioral Change of Addictive Behavior [45]. Emphasis is placed initially on feedback, future planning, and motivation for change, followed by reinforcement of progress and providing an objective perspective on the process of change. Collaboration with the client, versus confrontation or maintaining an authoritative approach, is a key strategy to this modality, providing opportunity for exploration as well. It is believed that change is motivated by a perceived discrepancy between present behavior and important personal goals and/or values, and so assisting the individual to identify such discrepancy is likely to facilitate change. Finally, supporting the individual to achieve self-efficacy around changing behaviors is a necessary principle of MI/ME.

In addition to incorporating MI/ME strategies throughout STRRP, psychoeducational information regarding mTBI and the impact of it when combined with SUDS and psychiatric disorders are provided. Psychoeducational materials were created from health and wellness models, and TBI-specific materials were adapted from a community-based intervention called the Substance Abuse and Traumatic Brain Injury Toolbox [46]. The toolbox was designed for healthcare facilities to address SA in clients receiving TBI rehabilitation in part by utilizing group media-based psychoeducation (e.g., a video) to address and facilitate substance use prevention through willingness to change behavior. Topics discussed include prevention, intervention, and treatment resources.

As currently conceptualized, STRRP is a manualized, 13-week, group-based intervention. Ideally, patients would be referred to treatment upon identification of positive mTBI history and/or persistent complications related to mTBI. Treatment topics emphasize recovery, resumption of work/social/interpersonal obligations, and intervention and prevention regarding risk-taking behaviors (e.g., excessive substance use, behaviors that may lead to reinjury or poor psychiatric outcomes). The intervention is highly experiential, and clients practice and consolidate their learning via weekly assignments.

## 4. Conclusions

Substance use and abuse frequently occurs with other risk-taking behaviors. While some interventions specifically may help decrease substance misuse, other interventions may be better suited to address additional risk-taking behaviors. Further, considerations for treatment regarding potential complications associated with history of mTBI are warranted, for example, when behavior or cognitive ability conflicts with ability to engage in or gain from the current treatment [47]. Strategies which accommodate for limitations in thinking (e.g., repetition of material and assignments in-between session for consolidation) may be indicated. Regardless of history of mTBI, however, the aim of treatment in any setting is likely to optimize functioning and quality of life. It would be expected that some benefit would result from addressing risk factors and possible sequelae associated with co-occurring mTBI and SUDs.

In any case, rigorous evaluation of potentially efficacious treatments addressing these co-occurring behaviors is missing in the literature. As such, the authors of this paper seek to evaluate the efficacy and effectiveness of STRRP via a sequential and orderly research plan starting with assessment of feasibility and establishing an evidence base. In addition to collecting data regarding outcomes of interest (e.g., patient readiness to change) practical feasibility-related questions such as the following must be addressed: (1) Will the intervention work given the structure and rules of an existing SUDs treatment program? (2) Will changes need to be made to the manual to better tailor the intervention to the target population? and (3) Will activities of the intervention interfere or conflict with the established services? Areas of interest include patient comprehension of outlined procedures and clinicians' ability to implement practices. Such procedures for this evaluation fall under “Stage 1” of Onken et al.'s model, [48, 49] which consists of pilot/feasibility testing, manual and training program development, and adherence/competence measurement for new and untested treatments. Areas of interest include patient comprehension of outlined procedures and clinicians' ability to implement practices. In line with the model [48], the proposed next steps may include modifications to content and/or procedures specified in the original treatment manual as a result of feedback and expert consultation throughout the duration of the proposed feasibility study. The result of feasibility testing ultimately will be a treatment and accompanying manual sufficiently prepared for future randomized clinical trials, and dissemination, and implementation research.

 In conclusion, further study is needed to clarify the mental health needs of individuals with co-occurring mTBI and SUDS to identify best practices. This proposed model may be a viable treatment approach that will contribute to the literature and evidence base. It may also offer significant impact in its attempt to address and improve significant clinical issues in military personnel and Veterans (co-occurring mTBI, SUD, and other risky behaviors).
